# New Treatment of Acute Rheumatism

**Published:** 1868-12

**Authors:** 


### New Treatment of Acute Rheumatism.

At St. Mary's we have noted of late several patients recovering from acute rheumatism, and learned something of the plan which has been adopted by Dr. Sibson during the last year in all cases without exception. It may be described as embracing three points. 1. Removal of pressure and tension of joints. 2. An even and warm temperature. 3. Removal or relief of pain. To accomplish the first of these ends, the patient lies in bed, and his joints are muffled in cotton, wool and flannel, a cradle being placed where the weight of the bed-clothes is painful. For the second, the patient wears a flannel dressing-gown, and the blankets touch the skin of the lower extremities, sheets being placed only over the upper part of the bed. For the third, the linimentum belladonna

[℞. Ext. Belladonna ℥j, Linim. Saponis f. ℥ viij. Ed.] is applied to painful joints, and covered over with wadding. Occasionally, where the pain is very excessive, from an eighth to a quarter of a grain of morphia is injected subcutaneously. For the rest, he has now and then found it useful to apply a leech or two to a swollen joint or to the cardiac region. In cases where there appears to be a gouty complication, Dr. Sibson employs a little iodide of potassium; but apart from this he does not give any potash to his patients. He tells us, in answer to an inquiry, that he finds the urine rarely containing acid after the first few days of treatment. As regards food, his experience and practice are not a little interesting. The patient is allowed from the first, roast meat, rice pudding and porter. We ascertained, moreover, from inquiry of the nurses, that this diet was not only ordered by the doctor, but was consumed by the patient, with very rare exceptions. Some patients to whom we spoke confirmed the statement; and added, also, strong testimony to the immense relief derived from the application of belladonna in the way described. The nurses said that the patient generally slept well at night.—*Lancet.*

